# Lenvatinib Plus Camrelizumab vs. Lenvatinib Monotherapy as First-Line Treatment for Unresectable Hepatocellular Carcinoma: A Multicenter Retrospective Cohort Study

**DOI:** 10.3389/fonc.2022.809709

**Published:** 2022-02-24

**Authors:** Qi Li, Mengran Cao, Guosheng Yuan, Xiao Cheng, Mengya Zang, Ming Chen, Xiaoyun Hu, Jing Huang, Rong Li, Yabing Guo, Jian Ruan, Jinzhang Chen

**Affiliations:** ^1^State Key Laboratory of Organ Failure Research, Guangdong Provincial Key Laboratory of Viral Hepatitis Research, Department of Infectious Diseases, Nanfang Hospital, Southern Medical University, Guangzhou, China; ^2^Department of Medical Oncology, Jinling Hospital, Medical School of Nanjing University, Nanjing University, Nanjing, China; ^3^Zengcheng Branch of Nanfang Hospital, Southern Medical University, Guangzhou, China; ^4^Department of Medical Oncology, The First Affiliated Hospital, College of Medicine, Zhejiang University, Hangzhou, China

**Keywords:** hepatocellular carcinoma, unresectable, lenvatinib, camrelizumab, objective response, survival

## Abstract

**Background:**

Combining an antiangiogenic agent with an anti-PD-1 agent is a promising strategy for unresectable hepatocellular carcinoma (HCC).

**Aims:**

To explore the effectiveness and tolerability of lenvatinib plus camrelizumab vs. lenvatinib monotherapy as a first-line treatment for unresectable HCC.

**Methods:**

This multicenter, retrospective cohort study included patients with unresectable HCC treated with oral lenvatinib 8 mg daily and intravenous camrelizumab 200 mg every 3 weeks (L+C group) or lenvatinib 12 mg or 8 mg daily (L group) in four Chinese centers between September 2018 and February 2020. Tumor response was evaluated according to RECIST 1.1 and mRECIST. The outcomes included objective response rate (ORR), overall survival (OS), 1-year OS rate, progression-free survival (PFS), and safety.

**Results:**

By March 31, 2021, 92 patients were finally included, with 48 and 44 in the L+C and L groups, respectively. ORR was significantly higher in the L+C group than in the L group (RECIST 1.1: 37.5% vs. 13.6%, P=0.009; mRECIST: 41.7% vs. 20.5%, P=0.029). Median OS and 95% confidence interval (CI) was 13.9 (13.3-18.3) months in the L group and not reached in the L+C group (P=0.015). The 1-year survival rate was 79.2% and 56.8% in the L+C and L groups, respectively. Median PFS was 10.3 (6.6-14.0) months and 7.5 (5.7-9.3) months in the L+C and L groups, respectively (P=0.0098). Combined therapy vs. monotherapy was independently associated with a prolonged OS (hazard ratio=0.380, 95% CI=: 0.196-0.739, P=0.004) and a prolonged PFS (hazard ratio=0.454, 95%CI=0.282-0.731, P=0.001). The safety profile was comparable between the two groups. The most common adverse event in the L+C and L groups was loss of appetite (41.7% vs. 40.9%, P=0.941). Three patients in the L+C group and two in the L group terminated treatment owing to adverse events.

**Conclusion:**

First-line lenvatinib plus camrelizumab showed better effectiveness than lenvatinib alone in patients with unresectable HCC.

## Introduction

Hepatocellular carcinoma (HCC) is a highly lethal invasive cancer arising in the liver (1, 2). The most important risk factors for HCC are infection with hepatitis B virus (HBV) or hepatitis C virus and/or preexisting liver cirrhosis (1–4). The worldwide age-standardized annual mortality rates for liver cancer are 13.9 per 100,000 men and 4.9 per 100,000 women (5, 6). HCC is typically asymptomatic throughout the initial clinical course of the disease (1, 4); hence about 50% of patients have advanced HCC at diagnosis (6). The 5-year overall survival (OS) of HCC is 18% for all stages, 31% for localized disease, 11% for regional disease, and only 2% for late-stage disease (5).

Although sorafenib has been used for many years as the first-line monotherapy for HCC, its use is associated with limited improvement in the prognosis of advanced HCC, and the newer option of lenvatinib provides better clinical benefits for patients with advanced HCC (7–10). The median progression-free survival (PFS) of patients with unresectable HCC treated with lenvatinib as a first-line monotherapy was 7.4 months, and the median OS was 13.6 months for lenvatinib compared with 12.3 months for sorafenib (7). Additionally, the objective response rate (ORR) was higher for lenvatinib than for sorafenib according to RECIST1.1 (24.1% vs. 9.2%) and mRECIST (40.6% vs. 12.4%) criteria. Nevertheless, further improvements in efficacy are required. Recent studies showed that a lenvatinib-based combination with immunotherapy could achieve better efficacy (11, 12). Combining antiangiogenic agents with immune checkpoint inhibitors has been a major breakthrough for the first-line treatment of HCC. Although atezolizumab plus bevacizumab as a first-line regimen for unresectable HCC resulted in better OS and PFS than treatment with sorafenib alone (13, 14), such combination therapy is quite expensive and not accessible to all patients.

*In vitro* studies have shown that lenvatinib and PD-1 inhibitors can exert synergistic antitumor effects, including activation of effector T cells and depletion of regulatory T cells in the tumor microenvironment, modulation of antigen-presenting cells and dendritic cell maturation, inhibition of immune-suppressive signaling, and normalization of tumor blood vessels (15–20). Furthermore, a retrospective analysis of first-line lenvatinib plus various PD-1 inhibitors in patients with unresectable HCC demonstrated tumor responses (21). A recent phase Ib study of lenvatinib plus pembrolizumab as first-line therapy for unresectable HCC provided preliminary evidence that combining an antiangiogenic agent with a PD-1 inhibitor exerted good antitumor activity against unresectable HCC (12). Another phase Ib study reported an ORR of 76.7% in patients with unresectable HCC treated with lenvatinib plus nivolumab (11). Lenvatinib is already covered by the medical insurance catalog for the treatment of HCC in China and has been widely applied in clinical practice. Therefore, studies are merited to investigate the effects of lenvatinib plus a PD-1 inhibitor as first-line therapy for patients with unresectable HCC.

Camrelizumab is a PD-1 inhibitor effective as a second-line treatment for HCC (22), and this agent has been approved for use in China. A retrospective study of patients with HCC who had received second-line therapy demonstrated that treatment with lenvatinib plus camrelizumab achieved longer survival than monotherapy with lenvatinib (23). Additionally, lenvatinib plus various PD-1 inhibitors with or without hepatic artery infusion chemotherapy (HAIC) was an effective first-line therapy for patients with advanced HCC (24). However, there remains no high-level evidence to guide drug selection among the available PD-1 inhibitors.

As mentioned above, the effectiveness and tolerability of lenvatinib plus camrelizumab as a first-line therapy still remain unclear. Therefore, this multicenter retrospective cohort study aimed to compare the therapeutic benefits and adverse reactions between lenvatinib plus camrelizumab and lenvatinib alone when given as a first-line treatment for patients with unresectable HCC.

## Materials and Methods

### Study Design and Patients

This multicenter retrospective cohort study included patients with unresectable HCC from four study centers in China (**Supplementary Table 1**) between September 2018 and February 2020. The inclusion criteria were: 1) diagnosed with HCC according to the Guidelines for the Diagnosis and Treatment of Primary Liver Cancer in China (2019 edition) (25); 2) Barcelona Clinic Liver Cancer (BCLC) stage B or C; 3) received lenvatinib plus camrelizumab or lenvatinib monotherapy as the first-line therapy; 4) Child-Pugh class A or B; 5) Eastern Cooperative Oncology Group performance score (ECOG PS) of 0-2; and 6) at least one measurable lesion as defined by the Response Evaluation Criteria in Solid Tumors (RECIST) 1.1 and modified Response Evaluation Criteria in Solid Tumors (mRECIST) 1.1. The exclusion criteria were: 1) concomitant other primary malignant tumors; 2) incomplete clinical data; 3) severe comorbidities such as heart disease, severe renal dysfunction or infection; 4) uncontrolled hypertension; 5) had undergone major surgery or experienced gastrointestinal hemorrhage within the previous 30 days; 6) pregnant or breastfeeding; 7) taking other antitumor agents; 8) total bilirubin >34.2 μmol/L, hepatic encephalopathy, or prolongation of prothrombin time (PT) >4 s; or 9) positive serology for hepatitis A, C or D or human immunodeficiency virus. This study was approved by the Ethics Committees of all four study centers. The requirement for individual informed consent was waived by the committees.

### Treatment and Follow-Up

The patients were divided into the lenvatinib plus camrelizumab and the lenvatinib monotherapy groups. Patients in the lenvatinib monotherapy group received oral lenvatinib (Eisai, Co., Ltd., Tokyo, Japan) with the dosage adjusted according to body weight (12 mg for patients ≥60 kg and 8 mg for patients <60 kg, once per day). Patients in the lenvatinib plus camrelizumab group received oral lenvatinib 8 mg daily and intravenous camrelizumab 200 mg every 3 weeks (Hengrui Medicine Co., Ltd., Jiangsu, China).

The treatment was discontinued if intolerable adverse events (AEs) or disease progression occurred. If lenvatinib administration had to be interrupted due to AEs, camrelizumab was not used alone during the discontinuation of lenvatinib owing to the high incidence of reactive cutaneous capillary endothelial proliferation. If the AE was related to camrelizumab and was confirmed to be an immune-related AE, camrelizumab was interrupted if the AE was of grade 2 or permanently stopped if the AE was of grade 3 or higher. If causality could not be determined between lenvatinib or camrelizumab, the administration of both drugs was interrupted if the AE was of grade 2 or permanently stopped if the AE was of grade 3 or higher.

Routine blood, liver function, renal function, and coagulation function tests, measurement of α-fetoprotein (AFP) level, enhanced computed tomography (CT), or enhanced magnetic resonance imaging (MRI) of the upper abdomen were performed every 6-8 weeks.

### Outcomes

The outcomes of this study included the ORR, disease control rate (DCR), OS, 1-year OS rate, PFS, and safety. An objective response was defined as a confirmed complete response (CR) or partial response (PR) according to RECIST and mRECIST 1.1. Disease control was defined as CR, PR, or stable disease (SD). The duration of treatment (DOT) was calculated. The time to response (TTR) was defined as the time from the start of treatment to the first confirmed CR or PR according to RECIST and mRECIST, respectively. OS was defined as the time from the start of treatment to death from any cause. PFS was defined as the time from the start of treatment to disease progression or death from any cause. The safety assessment included vital signs, hematological and biochemical laboratory tests, urinalysis, and electrocardiography. AEs were graded according to the National Cancer Institute Common Terminology Criteria for Adverse Events (NCI CTCAE) version 4.03.

### Statistical Analysis

All statistical analyses were performed using SPSS version 25.0 (IBM Corp., Armonk, NY, USA). Continuous data with a normal distribution are presented as means ± standard deviations and were compared using the independent t-test. Continuous data with a skewed distribution are presented as medians (ranges) and were analyzed with the Mann-Whitney U-test. Categorical data are presented as numbers (percentages) and were compared with the chi-squared test or Fisher’s exact test. The Kaplan-Meier method was used to calculate the survival time and plot the curve, and the log-rank test was used to compare the two groups. Multivariable Cox regression was used to explore the factors related to OS and PFS, including therapy used, body mass index, ECOG PS, Child-Pugh class, AFP level, tumor number, BCLC stage, HBV infection, vascular invasion, intrahepatic metastasis, extrahepatic metastasis, hand-foot syndrome, hypertension, proteinuria, and dysphonia. The variables with P<0.10 in the univariable analyses were included in the multivariable analysis. Two-sided P-values <0.05 were considered statistically significant.

## Results

### Study Population and Baseline Characteristics

**Figure 1:**
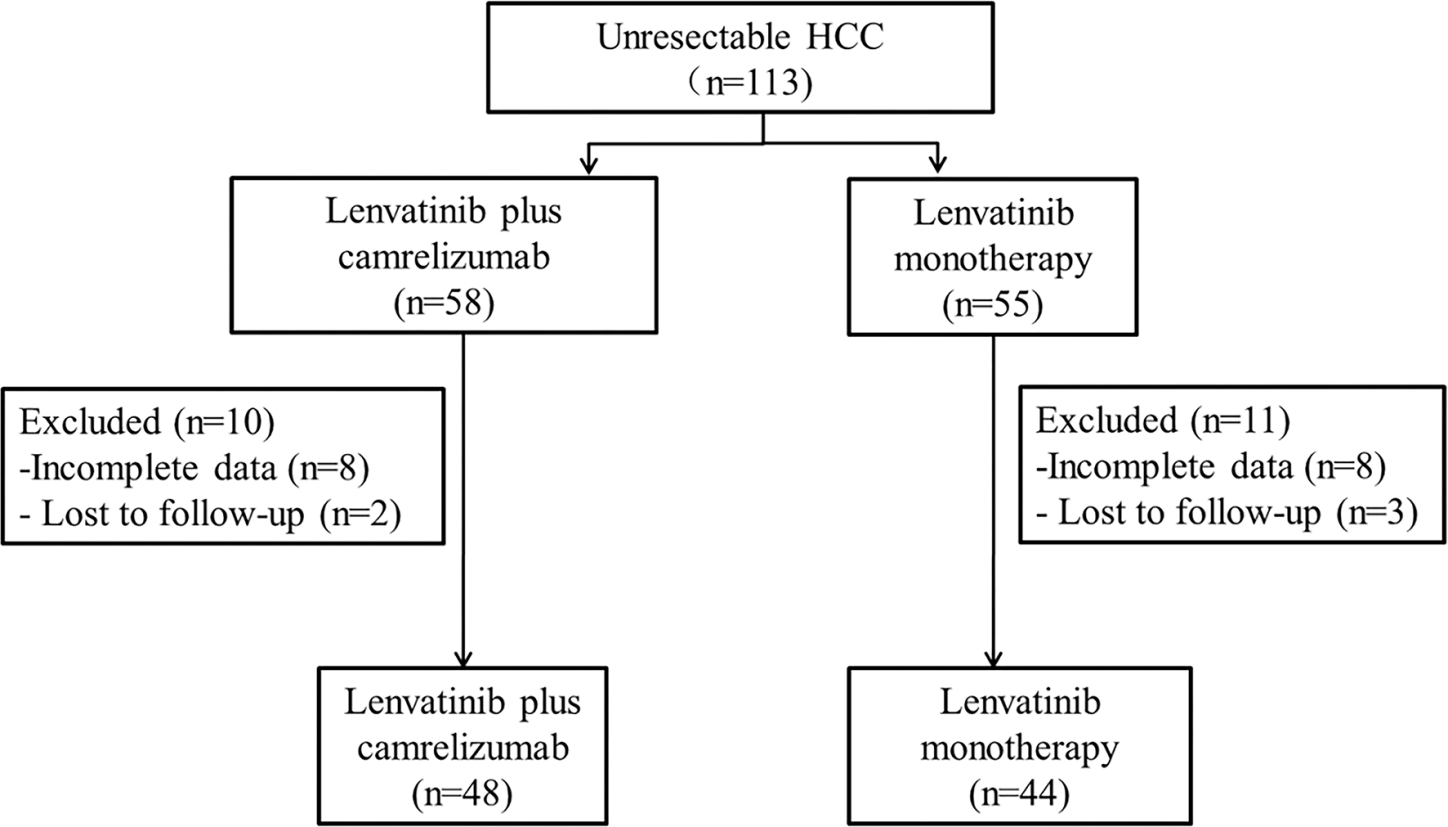
Patient flowchart.

Between September 2018 and February 2020, 113 patients with unresectable HCC in the four centers met the eligibility criteria (lenvatinib plus camrelizumab: n=58; lenvatinib monotherapy: n=55), but 21 patients were excluded. By the last follow-up on March 31, 2021, 92 patients were analyzed, of which 44 and 48 were in the lenvatinib plus camrelizumab and lenvatinib monotherapy groups, respectively (**Figure 1** and **Supplementary Table 1**). There were no significant differences between the two groups in the baseline clinical characteristics and previous treatments, including surgery and other treatments for HCC (all P>0.05; **Table 1**).

**Table 1:** Characteristics of the patients.

Characteristics	Lenvatinib plus camrelizumab group (n = 48)	Lenvatinib monotherapy group (n = 44)	P
Age (years)	53.81 ± 15.75	54.86 ± 18.25	0.692
Sex, male, n (%)	43 (89.6)	40 (90.9)	0.831
BMI (kg/m^2^)	22.33 ± 2.89	22.66 ± 3.09	0.600
ECOG PS, n (%)			0.984
0	20 (41.7)	18 (40.9)	
1	21 (43.8)	19 (43.2)	
2	7 (14.6)	7 (15.9)	
Platelets (×10^9^/L)	193.60 ± 86.13	187.77 ± 81.80	0.740
Total bilirubin (μmol/L)	18.72 ± 9.21	18.91 ± 8.89	0.920
Albumin (g/L)	36.79 ± 6.91	37.70 ± 6.45	0.504
Child-Pugh score, n (%)			0.752
≤7	41 (85.4)	40 (90.9)	0.417
>7	7 (14.6)	4 (9.1)	
AFP, n (%)			0.513
≤200 ng/mL	24 (50.0)	25 (56.8)	
>200 ng/mL	24 (50.0)	19 (43.2)	
Maximal diameter of tumor (cm)	9.95 ± 6.9	9.28 ± 4.6	0.508
Number of tumors, n (%)			0.638
≤3	9 (18.8)	10 (22.7)	
>3	39 (81.2)	34 (77.3)	
BCLC stage, n (%)			0.639
B	6 (12.5)	7 (15.9)	
C	42 (87.5)	37 (84.1)	
Vascular cancerous emboli, n (%)	36 (75)	34 (77.3)	0.799
Intrahepatic metastasis, n (%)	37 (80.4)	34 (77.3)	0.713
Distant metastasis, n (%)	21 (43.8)	19 (43.2)	0.956
ALBI, n (%)			0.707
1	16 (33.3)	17 (38.6)	
2	28 (58.3)	25 (56.8)	
3	4 (8.3)	2 (4.5)	
HBV infection, n (%)	41 (85.4)	38 (86.4)	0.896
Received previous treatment for HCC or not			0.452
Yes	32 (66.7%)	26 (59.1%)	
No	16 (33.3%)	18 (40.9%)	
Previous treatment(s) for HCC			
Surgery	11 (22.9%)	10 (22.7%)	0.983
Ablation	10 (20.1%)	9 (20.5%)	0.964
TACE or TAE	14 (29.2%)	10 (22.7%)	0.482

BMI, body mass index; ECOG PS, Eastern Cooperative Oncology Group performance status; AFP, α-fetoprotein; BCLC, Barcelona Clinic Liver Cancer; ALBI, assessment of the albumin-bilirubin; HBV, hepatitis B virus; HCC, hepatocellular carcinoma; TAE, transarterial embolization; TACE, transarterial chemoembolization.

### Effectiveness

**Figure 2:**
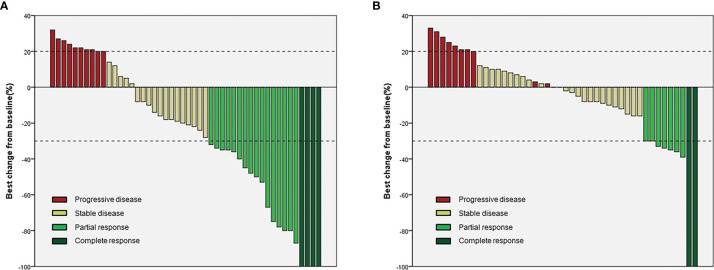
Waterfall plots for the two groups. **(A)** Lenvatinib plus camrelizumab; **(B)** Lenvatinib.

In the lenvatinib plus camrelizumab group, four patients achieved CR, 16 patients achieved PR, 18 patients had SD, and 10 patients had progressive disease (PD). In the lenvatinib monotherapy group, two patients achieved CR, seven patients achieved PR, 24 patients had SD, and 11 patients had PD (**Table 2** and **Figure 2**). ORR was significantly higher in the lenvatinib plus camrelizumab group than in the lenvatinib monotherapy group (RECIST 1.1: 37.5% vs. 13.6%, P=0.009; mRECIST: 41.7% vs. 20.5%, P=0.029). The DCR was not significantly different between the two groups (RECIST 1.1: 75.0% vs. 75.0%, P>0.999; mRECIST: 79.2% vs. 75.0%, P=0.634; **Table 2**). The DOT was significantly longer in the lenvatinib plus camrelizumab group than in the lenvatinib monotherapy group (10.45 [7.25-15.47] months vs. 7.5 [5.1-11.35] months, P=0.009). The TTR was similar between the two groups (RECIST 1.1: 6.27 [4.13-7.43] vs. 4.13 [3.38-5.48], P=0.068; mRECIST: 4.13 [3.37-5.4] vs. 3.6 [2.08-4.61], P=0.172) (**Table 2**).

**Table 2:** Treatment effects.

N (%)	RECIST 1.1			mRECIST		
	Lenvatinib plus camrelizumab (n = 48)	Lenvatinib monotherapy (n = 44)	P	Lenvatinib plus camrelizumab (n = 48)	Lenvatinib monotherapy (n = 44)	P
CR	2 (4.2%)	2 (4.5%)	0.031	4 (8.3%)	2 (4.5%)	0.177
PR	16 (33.3%)	4 (9.1%)		16 (33.3%)	7 (15.9%)	
SD	18 (37.5%)	27 (61.4%)		18 (37.5%)	24 (54.5%)	
PD	12 (25.0%)	11 (25.0%)		10 (20.8%)	11 (25.0%)	
ORR	18 (37.5%)	6 (13.6%)	0.009	20 (41.7%)	9 (20.5%)	0.029
DCR	36 (75.0%)	33 (75.0%)	>0.999	38 (79.2%)	33 (75.0%)	0.634
DOT	10.45 (7.25-15.47)	7.5 (5.1-11.35)	0.009			
TTR	6.27 (4.13-7.43)	4.13 (3.38-5.48)	0.068	4.13 (3.37-5.4)	3.6 (2.08-4.61)	0.172

Data are expressed as frequency (percentage). RECIST, Response Evaluation Criteria in Solid Tumor; mRECIST, modified Response Evaluation Criteria in Solid Tumor; CR, complete response; PR, partial response; SD, stable disease; PD, progressive disease; ORR, objective response rate; DCR, disease control rate; DOT, duration of treatment; TTR, time to response.

**Figure 3:**
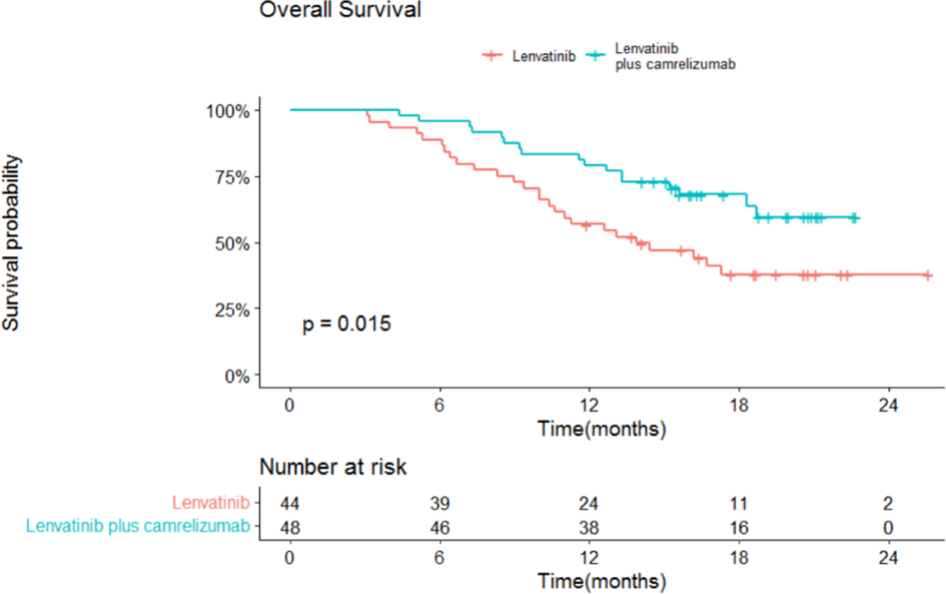
Overall survival (OS) in the two groups.

**Figure 4:**
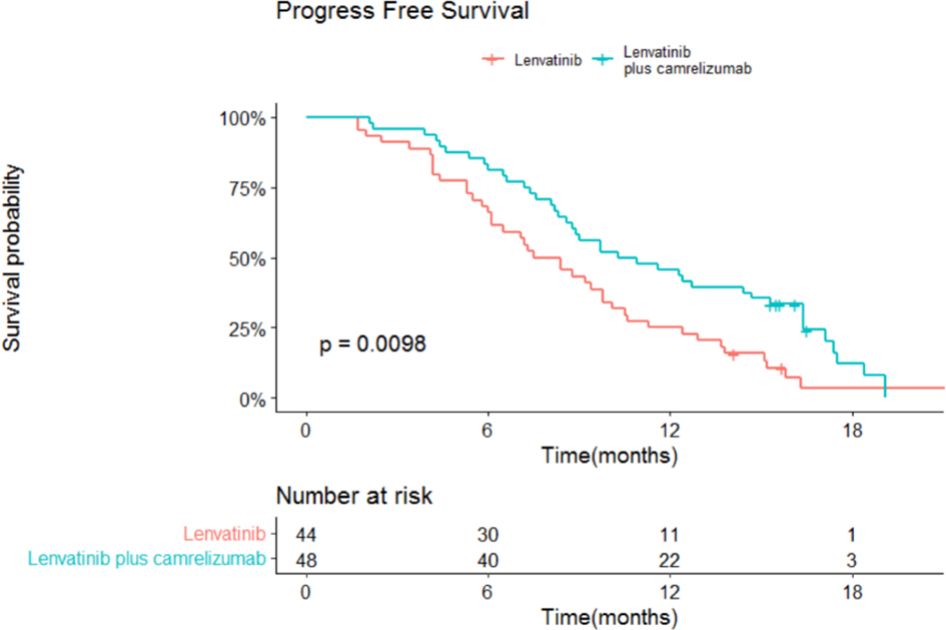
Progression-free survival (PFS) in the two groups.

The median OS and 95% confidence interval (CI) was 13.9 (13.3-18.3) months in the lenvatinib monotherapy group, while the median OS was not reached in the lenvatinib plus camrelizumab group (P=0.015; **Figure 3**). The 1-year survival rate was 79.2% in the lenvatinib plus camrelizumab group and 56.8% in the lenvatinib monotherapy group. The median PFS was significantly longer in the lenvatinib plus camrelizumab group than in the lenvatinib monotherapy group (10.3 [6.6-14.0] months vs. 7.5 [5.7-9.3] months, P=0.0098; **Figure 4**).

**Figure 5:**
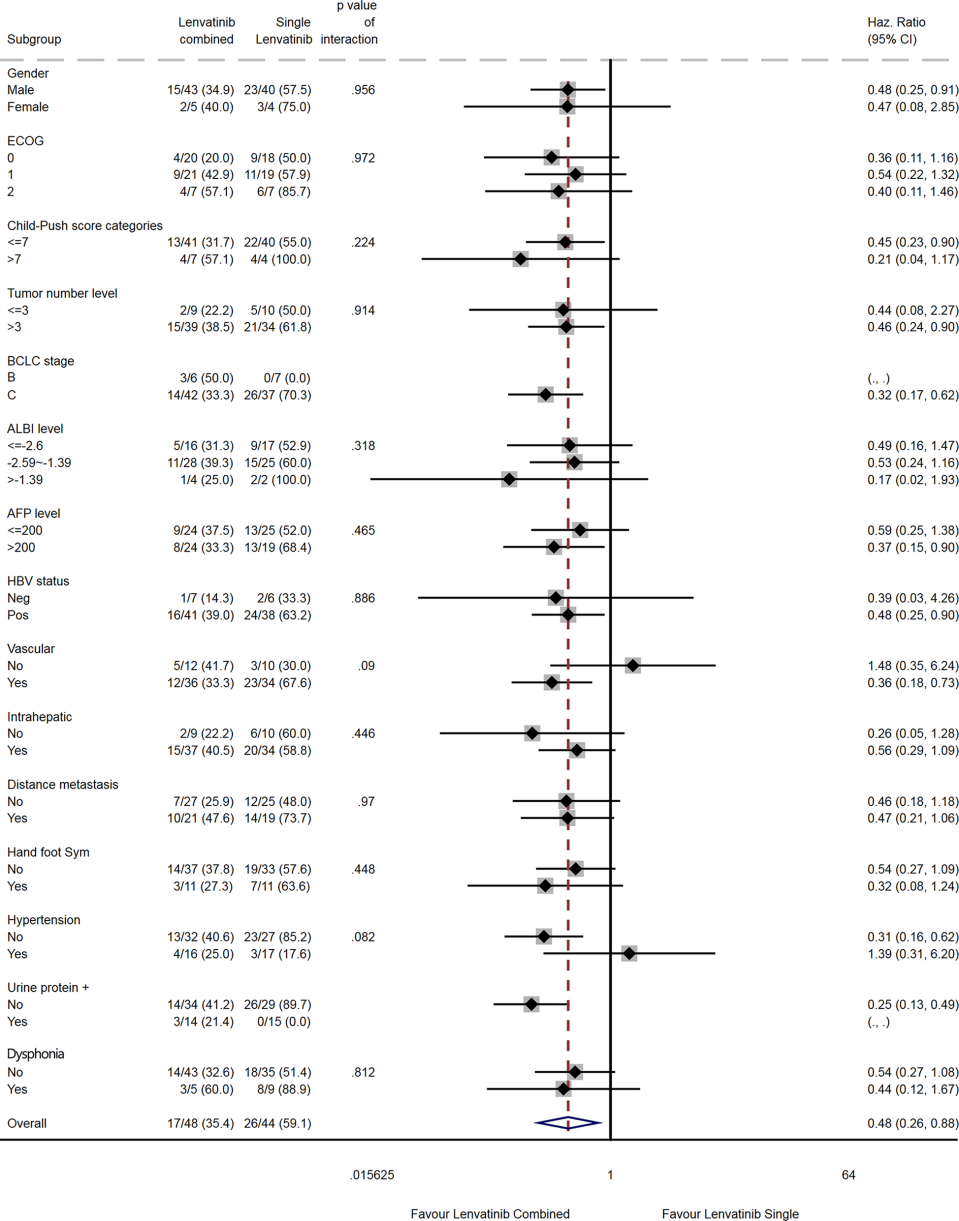
Subgroup analysis of overall survival.

**Figure 6:**
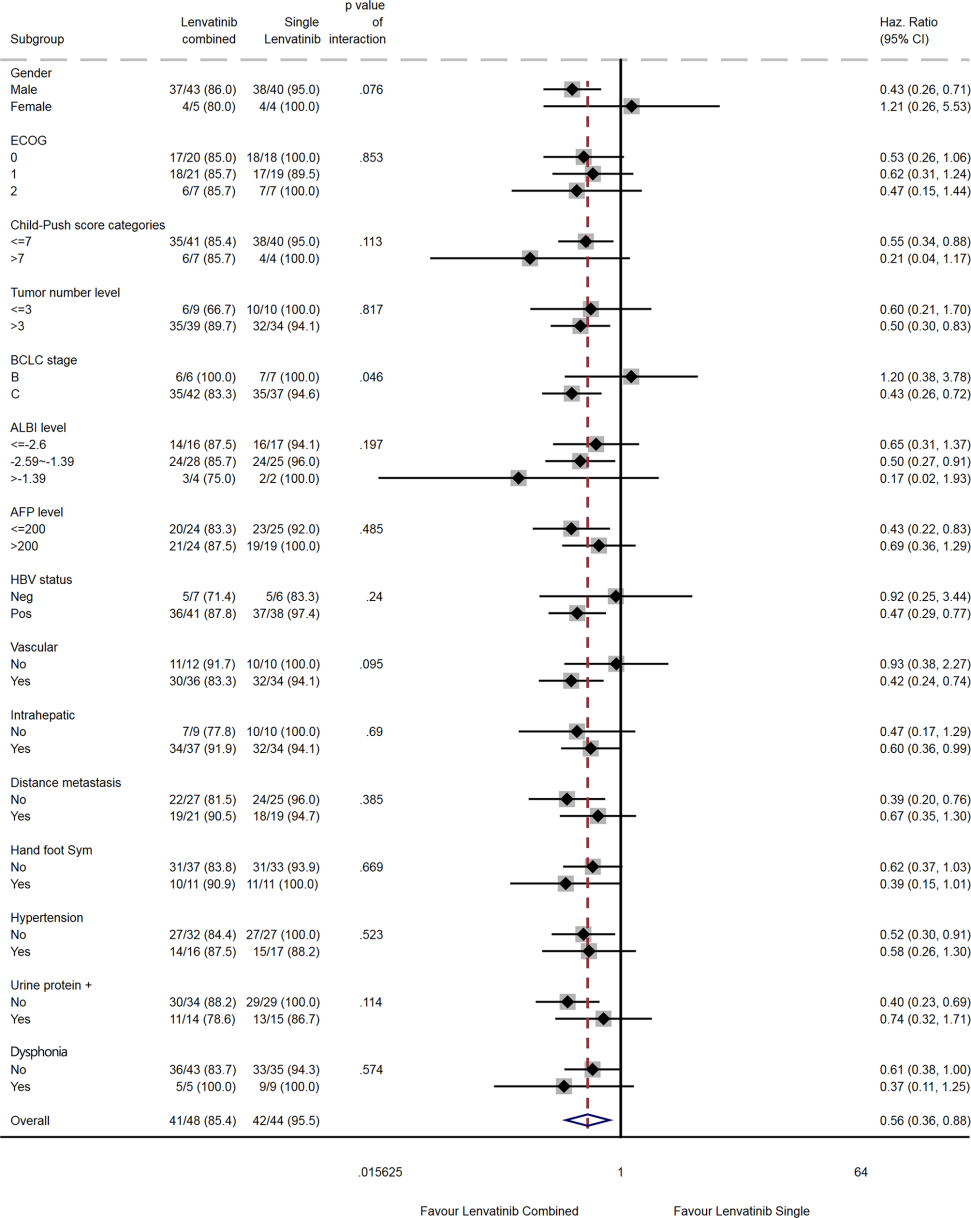
Subgroup analysis of progression-free survival.

The results of the subgroup analyses are shown in **Figures 5** and **6**. Compared with lenvatinib alone, combination therapy was associated with a prolonged OS in males (HR=0.48, 95% CI: 0.24-0.91), in patients with Child-Pugh score ≤7 (HR=0.45, 95% CI: 0.23-0.90), in patients with >3 tumors (HR=0.46, 95% CI: 0.24-0.90), in patients with AFP >200 ng/mL (HR=0.37, 95% CI: 0.15-0.90), in HBV-positive patients (HR=0.48, 95% CI: 0.25-0.90), in patients with vascular invasion (HR=0.36, 95% CI: 0.18-0.73), and in patients without hypertension (HR=0.31, 95% CI: 0.16-0.62; **Figure 5**). There were no differences among subgroups for ECOG PS score, albumin-bilirubin (ALBI) score, intrahepatic metastasis, distant metastasis, hand-foot syndrome, and dysphonia. Subgroup analyses for the BCLC stage and urinary proteins could not be performed because of a lack of events in one subgroup each.

Compared with lenvatinib alone, combination therapy was associated with a prolonged PFS in males (HR=0.43, 95% CI: 0.26-0.71), in patients with Child-Pugh score ≤7 (HR=0.55, 95%CI: 0.34-0.88), in patients with >3 tumors (HR=0.50, 95% CI: 0.30-0.83), in patients with BCLC stage C (HR=0.43, 95% CI: 0.26-0.72), in patients with an ALBI score of -2.59 to -1.39 (HR=0.50, 95% CI: 0.27-0.91), in patients with AFP ≤200 ng/mL (HR=0.43, 95% CI: 0.22-0.83), in HBV-positive patients (HR=0.47, 95% CI: 0.29-0.77), in patients with vascular invasion (HR=0.42, 95% CI: 0.24-0.74), in patients with intrahepatic metastasis (HR=0.60, 95% CI: 0.36-0.99), in patients without distant metastasis (HR=0.39, 95% CI: 0.20-0.76), and in patients without hypertension (HR=0.52, 95% CI: 0.30-0.91; **Figure 6**). There were no differences among subgroups for ECOG, hand-foot syndrome and dysphonia.

### Adverse Events

In the lenvatinib plus camrelizumab group, five patients (10.4%) had treatment-related AEs (TRAEs) leading to dose reduction, seven patients (14.6%) had TRAEs causing treatment suspension and three patients (6.3%) had TRAEs causing permanent termination of treatment. In the lenvatinib monotherapy group, six patients (13.6%) had TRAEs causing dose reduction, eight patients (18.2%) had TRAEs leading to treatment suspension, and two patients (4.5%) had TRAEs causing permanent termination of treatment.

The AEs which occurred in more than 20% of patients in the lenvatinib plus camrelizumab and lenvatinib monotherapy groups were hand-foot syndrome (22.9% vs. 25.0%, P=0.815), hypertension (33.3% vs. 38.6%, P=0.596), diarrhea (31.2% vs. 31.8%, P=0.953), loss of appetite (41.7% vs. 40.9%, P=0.941), proteinuria (29.2% vs. 34.1%, P=0.612) and increased alanine transaminase (22.9% vs. 25.0%, P=0.815). There were no statistically significant differences between the two groups in the incidences of any AEs (**Table 3**). The most common grade ≥3 AEs were hypertension (12.5% vs. 13.6%, P=0.872), proteinuria (4.2% vs. 4.5%, P=0.658), dysphonia (2.1% vs. 4.5%, P=0.467), diarrhea (2.1% vs. 2.3%, P=0.731), and increased ALT (2.1% vs. 2.3%, P=0.731).

**Table 3:** Adverse events of all grades and grade ≥3 in this study.

AE, n (%)	All grades			Grade ≥3		
	Lenvatinib plus camrelizumab (n = 48)	Lenvatinib monotherapy (n = 44)	P	Lenvatinib plus camrelizumab (n = 48)	Lenvatinib monotherapy (n = 44)	P
Hand-foot syndrome	11 (22.9%)	11 (25.0%)	0.815	0	1 (2.3%)	0.478
Hypertension	16 (33.3%)	17 (38.6%)	0.596	6 (12.5%)	6 (13.6%)	0.872
Diarrhea	15 (31.2%)	14 (31.8%)	0.953	1 (2.1%)	1 (2.3%)	0.731
Loss of appetite	20 (41.7%)	18 (40.9%)	0.941	0	1 (2.3%)	0.478
Proteinuria	14 (29.2%)	15 (34.1%)	0.612	2 (4.2%)	2 (4.5%)	0.658
Increased ALT	11 (22.9%)	11 (25.0%)	0.815	1 (2.1%)	1 (2.3%)	0.731
Thrombocytopenia	7 (14.6%)	7 (15.9%)	0.860	0	0	/
Dysphonia	5 (10.4%)	8 (18.2%)	0.285	1 (2.1%)	2 (4.5%)	0.467
Hypothyroidism	6 (12.5%)	6 (13.6%)	0.872	0	0	/

ALT, alanine transaminase.

### Multivariable Analysis of Factors Associated With OS and PFS

Cox regression analysis showed that combination therapy vs. monotherapy (HR=0.380, 95% CI: 0.196-0.739, P=0.004), ECOG PS 2 vs. 0 (HR=6.769, 95% CI: 2.183-20.989, P=0.001), hypertension (HR=0.393, 95% CI: 0.163-0.944, P=0.037), proteinuria (HR=0.196, 95% CI: 0.054-0.704, P=0.012), and dysphonia (HR=2.386, 95% CI: 1.022-5.57, P=0.044) were independently associated with a prolonged OS. (**Table 4**). Furthermore, combination therapy vs. monotherapy (HR=0.454, 95% CI: 0.282-0.731, P=0.001) and ECOG PS 2 vs. 0 (HR=2.955, 95% CI: 1.416-6.166, P=0.004) were independently associated with a prolonged PFS (**Table 5**).

**Table 4:** Multivariable Cox regression analysis for OS.

	Univariable analysis			Multivariable analysis		
	HR	95% CI	P	HR	95% CI	P
Combined therapy vs. monotherapy	0.477	0.259-0.881	0.018	0.380	0.196-0.739	0.004
Body mass index	0.917	0.823-1.021	0.112			
ECOG PS			<0.001			0.003
1 vs. 0	1.842	0.907-3.741	0.091	1.298	0.610-2.764	0.498
2 vs. 0	5.631	2.375-13.35	<0.001	6.769	2.183-20.989	0.001
Child-Pugh level (C vs. B)	2.496	1.363-4.571	0.003	1.405	0.516-3.821	0.506
AFP level (>200 vs. ≤200)	1.063	0.584-1.934	0.841			
Tumor number (>3 vs. ≤3)	1.441	0.641-3.241	0.377			
BCLC stage (C vs. B)	2.887	0.890-9.362	0.077	1.749	0.462-6.614	0.410
HBV infection	2.836	0.887-9.173	0.082	2.321	0.637-8.46	0.202
Vascular invasion (yes vs. no)	1.727	0.799-3.733	0.165			
Intrahepatic metastasis (yes vs. no)	1.521	0.705-3.281	0.285			
Extrahepatic metastasis (yes vs. no)	2.03	1.111-3.710	0.021	1.568	0.795-3.09	0.194
Hand-foot syndrome (yes vs. no)	0.932	0.459-1.893	0.847			
Hypertension (yes vs. no)	0.272	0.121-0.613	0.002	0.393	0.163-0.944	0.037
Proteinuria (yes vs. no)	0.11	0.034-0.355	<0.001	0.196	0.054-0.704	0.012
Dysphonia (yes vs. no)	2.5	1.221-5.119	0.012	2.386	1.022-5.57	0.044

HR, hazard ratio; CI, confidence interval; ECOG PS, Eastern Cooperative Oncology Group performance status; AFP, α-fetoprotein; BCLC, Barcelona Clinic Liver Cancer; HBV, hepatitis B virus.

**Table 5:** Multivariable Cox regression analysis for PFS.

	Univariable analysis			Multivariable analysis		
	HR	95% CI	P	HR	95% CI	P
Combined therapy vs. monotherapy	0.567	0.363-0.885	0.012	0.454	0.282-0.731	0.001
Body mass index	0.954	0.883-1.031	0.23			
ECOG PS			<0.001			
1 vs. 0	1.104	0.684-1.781	0.685	0.940	0.571-1.546	0.807
2 vs. 0	2.773	1.436-5.358	0.002	2.955	1.416-6.166	0.004
Child-Pugh level (C vs. B)	2.242	1.143-4.399	0.019	1.584	0.742-3.385	0.235
AFP level (>200 vs. ≤200)	1.019	0.659-1.574	0.934			
Tumor number (>3 vs. ≤3)	1.724	0.941-3.161	0.078			
BCLC stage (C vs. B)	1.097	0.592-2.035	0.768			
HBV infection	2.106	1.048-4.232	0.036	1.813	0.828-3.968	0.136
Vascular invasion (yes vs. no)	1.027	0.619-1.704	0.919			
Intrahepatic metastasis (yes vs. no)	1.869	1.058-3.300	0.031	1.494	0.820-2.723	0.190
Extrahepatic metastasis (yes vs. no)	1.347	0.869-2.088	0.183	1.568	0.795-3.09	0.194
Hand-foot syndrome (yes vs. no)	1.251	0.758-2.067	0.381			
Hypertension (yes vs. no)	0.868	0.551-1.368	0.542			
Proteinuria (yes vs. no)	0.557	0.340-0.913	0.020	0.598	0.335-1.067	0.082
Dysphonia (yes vs. no)	1.702	0.947-3.060	0.075	1.500	0.804-2.798	0.202

HR, hazard ratio; CI, confidence interval; ECOG PS, Eastern Cooperative Oncology Group performance status; AFP, α-fetoprotein; BCLC, Barcelona Clinic Liver Cancer; HBV, hepatitis B virus.

## Discussion

This multicenter retrospective cohort study compared treatment responses and adverse events between lenvatinib plus camrelizumab and lenvatinib alone, given as the first-line treatment for unresectable HCC. The findings suggest that treatment with the combination of lenvatinib and camrelizumab might improve the ORR, PFS, and OS of patients when compared with lenvatinib monotherapy. The toxicity profile and tolerance were similar between the two groups, and no new safety signals were identified.

The combination of lenvatinib with a PD-1 inhibitor has been used in various solid cancers (26, 27), including HCC (12, 28–30), cholangiocarcinoma (31, 32), renal cancer (33–37), endometrial cancer (38–41), gastric cancer (42, 43) and adrenal cortical carcinoma (44). Lenvatinib plus a PD-1 inhibitor appears to be effective in patients with corresponding molecular subtypes regardless of the type of cancer, and the evidence from clinical trials indicates that the effectiveness of this treatment regimen might depend on the molecular subtype rather than the type of cancer. Hence, lenvatinib combined with an anti-PD-1 agent might be a promising option for many solid tumors.

The OS, PFS, and ORR (mRECIST) for patients in the lenvatinib monotherapy group were 13.9 months, 7.5 months, and 20.5%, respectively, in agreement with a previous study that reported corresponding values of 13.6 months, 7.4 months, and 24.1%, respectively, in patients with unresectable HCC (7). The median PFS for patients in the lenvatinib plus camrelizumab group was 10.3 months, the ORR (mRECIST) was 41.7%, and data for the estimation of OS were immature, indicating that the overall treatment benefits were greater in the lenvatinib plus camrelizumab group than in the lenvatinib monotherapy group. Our findings are supported by Wei et al. (23), who reported higher ORR and DCR for lenvatinib plus camrelizumab than for lenvatinib alone when used as second-line therapy. Additionally, a previous case report presented a patient with gastric cancer and liver metastasis who remained progression-free after 14 months of treatment with lenvatinib and camrelizumab (45). The above results may be associated with synergistic effects between the two types of immunotherapy, as suggested by *in vitro* experiments (15, 16), a retrospective study of lenvatinib combined with various anti-PD-1 therapies (21), and a phase Ib clinical trial (12). The exact mechanisms underlying this synergy remain uncharacterized. Besides its antiangiogenic actions, lenvatinib also modulates the immune system and reverses immunosuppression by promoting dendritic cell maturation, increasing the proliferation, tumor infiltration, and antitumor activity of effector T cells, upregulating T cell-related chemokines in the tumor, reducing the number of regulatory T cells, and inhibiting myeloid-derived suppressor cells (17). In the context of immune upregulation, inhibiting immune checkpoints might strengthen antitumor immunity (46, 47). Indeed, anti-PD-1 agents enhance tumor infiltration by dendritic cells and effector T cells (18), which would augment similar actions exerted by lenvatinib. Furthermore, inhibition of CTLA-4, another immune checkpoint, depletes regulatory T cells and thus reduces the degree of immunosuppression in the tumor microenvironment (19). Interestingly, the combination of lenvatinib with a PD-1 inhibitor attenuate immunosuppressive mechanisms and create an immune-active microenvironment substantially, and these effects were greater than those seen for each agent alone (18). Additionally, combining an inhibitor of vascular endothelial growth factor with a checkpoint inhibitor improved the migration of antigen-specific T cells (20). It has been reported that the use of multi-targeted tyrosine kinase inhibitor regorafenib combined with anti-PD-1 therapy in HCC could have a synergistic antitumor effect that is worth exploring, since regorafenib might modulate macrophage polarization, increase T cell activation, and thus enhance the efficacy of anti-PD-1 therapy (48). The concept of synergism is further supported by a meta-analysis concluding that lenvatinib plus pembrolizumab achieved better treatment outcomes than lenvatinib alone or pembrolizumab alone (49). Such combinations might act on both the vasculature and the stimulation of the antitumor immunity. Still, trials will have to examine these combinations.

Prior clinical investigations have suggested that the response to lenvatinib was smaller in patients with a high disease burden (50) or impaired liver function (51). One of the strengths of the present study is that it included many patients with late-stage liver cancer and thus reflects the situation encountered in real-world clinical practice. Thus, our investigation has a notable advantage over previous studies of lenvatinib as first-line therapy for HCC, including fewer patients with late-stage HCC. For example, the REFLECT trial, which compared lenvatinib monotherapy with sorafenib monotherapy, excluded patients categorized with Child-Pugh class B and ECOG PS score of 2 (7), whereas our study included such patients. Similarly, a recent retrospective analysis of 41 patients with advanced HCC included only one patient with an ECOG PS score of ≥1, and extrahepatic metastasis was present in only 24% of cases (52), compared with 43% in our study. Notably, a retrospective study of patients not meeting the REFLECT trial eligibility criteria concluded that the efficacy of lenvatinib was comparable between patients with/without Child-Pugh class B and between patients with/without tumor in ≥50% of the liver (53), suggesting that lenvatinib remains effective in those with more advanced disease. Similarly, our study revealed very promising results for OS, PFS, and ORR in patients treated with combination therapy despite including many cases with late-stage HCC. Hence, our findings provide indirect evidence that first-line treatment with lenvatinib and camrelizumab might benefit patients with unresectable HCC in a real-world clinical setting.

Targeted immunotherapy greatly improves the ORR of advanced HCC. Combining anti-angiogenic drugs with immunotherapy for advanced or unresectable HCC can achieve an ORR of about 30%, and the median survival time of the patients can be increased to about 20 months (12, 13). By comparison, the median postoperative survival is only 12-15 months when surgical treatment is considered the first choice for HCC with resectable intrahepatic lesions and vascular invasion (i.e., technically resectable CNLC stage IIIa disease) (54). With the progress of drug treatment, many investigators began to explore the combination of target therapy and immunotherapy to reduce the tumor load for CNLC stage IIB and IIIA HCC, improve the R0 resection rate, and reduce the surgical risk, or resect the tumor after downstaging, to achieve better survival benefits than other treatments. Still, it is a retrospective cohort study with a small sample size (55). However, postoperative recurrence of HCC remains a major problem. Although the short-term remission rate was improved in patients with HCC who underwent surgical resection after targeted therapy and immunotherapy, relevant data about long-term OS are still lacking. Further research is needed to determine the optimal combination of drugs and the optimal time for surgical resection, develop methods of predicting the efficacy of combination therapy, and establish whether adjuvant therapy is necessary after surgical resection. As a result, many clinicians and patients still adopt a “wait-and-see” approach regarding combination therapy.

The incidence of AEs was relatively high in our study, but most AEs were manageable. The combined therapy did not significantly aggravate the incidence or severity of AEs compared with lenvatinib alone. The AEs were similar to those already reported for the two drugs (7, 12, 21–23), and no new safety signals were identified in this study. The treatments were well tolerated, with no grade 5 TRAEs. In agreement with our findings, a previous meta-analysis also showed that lenvatinib plus pembrolizumab had a similar safety profile to lenvatinib alone or pembrolizumab alone (49).

Beneficial clinical effects of transcatheter arterial chemoembolization (TACE) and HAIC have been demonstrated in patients with intermediate-stage liver cancer, but these treatment options are not suitable for those with late-stage disease (56, 57). The Chinese clinical guidelines for managing HCC recommend that patients with CNLC stage IIb/IIIa disease and some with stage IIIb disease are suitable for TACE and HAIC (58). However, there is evidence that multiple TACE procedures can cause an attenuation of the response and impairment of liver function (56). Interestingly, lenvatinib can suppress the development of liver fibrosis in preclinical experiments (59) and help maintain a liver functional reserve in the patients (60). Since the present study suggests that lenvatinib plus camrelizumab has promising efficacy in patients with intermediate-stage HCC, it will be worth exploring whether combining lenvatinib and camrelizumab with TACE or HAIC might have additional clinical benefits.

The use of camrelizumab monotherapy can lead to reactive cutaneous capillary endothelial proliferation (RCCEP), but the incidence of this adverse effect is decreased significantly if camrelizumab is combined with a targeted anti-angiogenic drug. Therefore, none of the patients in this study were treated with camrelizumab alone, and as a result, there were no cases of RCCEP. The multivariable regression analysis showed that, in addition to combination therapy, the ECOG PS score was associated with prognostic outcomes (OS and PFS). The ECOG PS score is a well-known prognostic factor in patients with cancer (61, 62). In addition, our findings showed that hypertension and proteinuria were associated with a longer OS, suggesting that the occurrence of hypertension and proteinuria might be indicators of good treatment response. Similar results were observed with bevacizumab in patients with glioblastoma (63) and antiangiogenic therapies in metastatic colorectal cancer (64). In HCC treated with sorafenib, the occurrence of off-target AEs including hypertension, diarrhea, skin toxicity, and fatigue have been shown to be positively related to better treatment response of time to progression and OS (65). Furthermore, some immune-related AEs with anti-PD-1 therapies have been associated with a good prognosis in patients with colorectal cancer and non-small cell lung cancer (66–68), but this association has not been previously reported for camrelizumab in patients HCC. Therefore, patients with such AEs should be managed appropriately and should be encouraged to continue treatment since these AEs might be predictive of treatment response. It will require further investigation in future studies.

The combination of bevacizumab and atezolizumab is expensive, with healthcare costs of $313,193 compared to $156,984 for sorafenib and an incremental cost-effectiveness ratio of $322,500 per quality-adjusted life-year (69). Therefore, many healthcare insurances do not reimburse the costs of this combination, and many patients cannot afford or have access to such a regimen. In China, lenvatinib is already covered by medical insurance as first-line therapy for HCC, and thus the accessibility of lenvatinib plus camrelizumab is higher. Additionally, camrelizumab has a remarkable price advantage in China (2928 RMB/cycle or USD 2300 a year), where it was developed and has been widely applied for cancer therapy, especially since it is covered by national medical insurance.

The subgroup analyses suggested that patients with specific characteristics might benefit more than others from the lenvatinib plus camrelizumab combination. However, the results of the subgroup analyses must be interpreted with caution because some subgroups were small and had few events. The study was not powered to reach firm conclusions about these subgroup analyses. Nevertheless, males, patients with Child-Pugh score ≤7, >3 tumors, AFP level >200 mg/dL, HBV infection or vascular invasion, and patients without hypertension might benefit more than their counterparts. Additional studies are needed to verify which patients might exhibit better outcomes after treatment with lenvatinib and camrelizumab. Moreover, large-scale studies comparing the therapeutic and adverse effects of different combinations of drugs in different patient subgroups might in the future allow for individualized therapies to be selected based on the clinical characteristics of the patient.

This study has limitations. It was a retrospective study with a relatively small sample size. The analyzable data were limited to those available in the medical charts. Furthermore, the follow-up was relatively short, and the data for several endpoints, such as OS, were still immature. Additional studies and randomized controlled trials should be performed to confirm these results. Such a trial (ClinicalTrials.gov NCT04443309) is currently underway.

In conclusion, first-line therapy with lenvatinib plus camrelizumab might benefit patients with unresectable HCC more than lenvatinib monotherapy. The toxicity profile and tolerability appeared similar between the two therapeutic regimens, and there were no new safety signals. Combined therapy with lenvatinib and camrelizumab might provide a new treatment option for patients with unresectable HCC and is worth further investigating.

## Data Availability Statement

The raw data supporting the conclusions of this article will be made available by the authors, without undue reservation.

## Ethics Statement

The studies involving human participants were reviewed and approved by The Ethics Committees of all four study centers. The requirement for individual informed consent was waived by the committees.

## Author Contributions

QL, MRC, and GY have full access to all of the data in the study and take responsibility for the integrity of the data and the accuracy of the data analysis. JC and JR were involved in the study conceptualization and design. All authors (QL, MRC, GY, XC, MZ, MC, XH, JH, YG, RL, JR, and JC) were involved in the acquisition, analysis, and interpretation of data. QL and JR supervised the analysis. QL and JC were involved in the drafting of the manuscript. All authors read, critically revised, and approved the manuscript.

## Funding

The study was partly supported by the Chen Xiao-Ping Foundation for the Development of Science and Technology (CXPJJH11900009-02), the Postdoctoral Research Foundation of China (No. 2021M691468), and the Special Fund for Clinical Research of the Nanfang Hospital, Southern Medical University (No. 2020CR019, No. 2020CR021).

## Conflict of Interest

The authors declare that the research was conducted in the absence of any commercial or financial relationships that could be construed as a potential conflict of interest.

## Publisher’s Note

All claims expressed in this article are solely those of the authors and do not necessarily represent those of their affiliated organizations, or those of the publisher, the editors and the reviewers. Any product that may be evaluated in this article, or claim that may be made by its manufacturer, is not guaranteed or endorsed by the publisher.
