# Supplementary material for: Lenvatinib Plus Camrelizumab vs. Lenvatinib Monotherapy as First-Line Treatment for Unresectable Hepatocellular Carcinoma: A Multicenter Retrospective Cohort Study
Source: Front Oncol. 2022 Feb 24;12:809709. doi: 10.3389/fonc.2022.809709 (PMC8907842; doi:10.3389/fonc.2022.809709)
Supplement: Supplementary file 1 [file Table_1.docx]

**Supplementary Table 1.** List of the study centers that participated in this study

| No. | Study center | Patients included (n) |
| --- | --- | --- |
| 1 | Nanfang Hospital of Nanfang Medical University | 55 |
| 2 | Zengcheng Branch of Nanfang Hospital, Nanfang Medical University | 7 |
| 3 | Affiliated Jinling Hospital, Medical School of Nanjing University, Jiangsu | 17 |
| 4 | The First Hospital, Zhejiang University School of Medicine | 13 |
| Total |  | 92 |
